# The Actin Binding Domain of βI-Spectrin Regulates the Morphological and Functional Dynamics of Dendritic Spines

**DOI:** 10.1371/journal.pone.0016197

**Published:** 2011-01-31

**Authors:** Michael W. Nestor, Xiang Cai, Michele R. Stone, Robert J. Bloch, Scott M. Thompson

**Affiliations:** 1 Department of Physiology, University of Maryland School of Medicine, Baltimore, Maryland, United States of America; 2 Program in Neuroscience, University of Maryland School of Medicine, Baltimore, Maryland, United States of America; 3 Training Program in Integrative Membrane Biology, University of Maryland School of Medicine, Baltimore, Maryland, United States of America; Medical College of Georgia, United States of America

## Abstract

Actin microfilaments regulate the size, shape and mobility of dendritic spines and are in turn regulated by actin binding proteins and small GTPases. The βI isoform of spectrin, a protein that links the actin cytoskeleton to membrane proteins, is present in spines. To understand its function, we expressed its actin-binding domain (ABD) in CA1 pyramidal neurons in hippocampal slice cultures. The ABD of βI-spectrin bundled actin in principal dendrites and was concentrated in dendritic spines, where it significantly increased the size of the spine head. These effects were not observed after expression of homologous ABDs of utrophin, dystrophin, and α-actinin. Treatment of slice cultures with latrunculin-B significantly decreased spine head size and decreased actin-GFP fluorescence in cells expressing the ABD of α-actinin, but not the ABD of βI-spectrin, suggesting that its presence inhibits actin depolymerization. We also observed an increase in the area of GFP-tagged PSD-95 in the spine head and an increase in the amplitude of mEPSCs at spines expressing the ABD of βI-spectrin. The effects of the βI-spectrin ABD on spine size and mEPSC amplitude were mimicked by expressing wild-type Rac3, a small GTPase that co-immunoprecipitates specifically with βI-spectrin in extracts of cultured cortical neurons. Spine size was normal in cells co-expressing a dominant negative Rac3 construct with the βI-spectrin ABD. We suggest that βI-spectrin is a synaptic protein that can modulate both the morphological and functional dynamics of dendritic spines, perhaps via interaction with actin and Rac3.

## Introduction

Most excitatory synapses are positioned on dendritic spines, which display a range of sizes and shapes. Changes in dendritic spine size accompany synapse maturation [1] and are correlated with synaptic strength [2]–[4]. Spine morphology is altered by changes in synaptic activity [5], [6] and can accompany changes in synaptic strength in some, but not all, forms of long-term potentiation [7]–[9]. Conversely, abnormal dendritic spines are observed in many forms of mental dysfunction. The signaling mechanisms that regulate spine structure and couple changes in synaptic structure and function are now being actively explored.

Actin, both G-actin monomers and filamentous, polymerized F-actin, is highly concentrated in dendritic spines [10] and regulates spine morphology. Dynamic changes in actin polymerization underlie morphological plasticity of spines [11], [12] and can affect synaptic function [10], [13]–[17]. Actin also serves as the scaffold to which transmembrane proteins like AMPA and NMDA receptors, as well as intracellular signaling molecules, are anchored [18]–[20]. Treatment with actin-depolymerizing agents reduces the number of functional AMPARs at dendritic spines [21], [22]. Thus, actin and its binding proteins, as well as the signaling molecules that regulate their interaction, are essential for synapse structure and function.

Spectrin is a large protein that links the actin cytoskeleton to the cytoplasmic surface of plasma membranes [23], [24] and regulates the dynamic state of the actin cytoskeleton [25]. Spectrin also anchors transmembrane proteins, such as NMDARs [26], to the actin cytoskeleton, generating localized microdomains that facilitate rapid signaling cascades. Spectrin is a heterodimer, consisting of α- and β-chains [27], [28]. βI-spectrin is enriched in postsynaptic spine heads in the absence of α-subunits [29]. Proteins in the spectrin superfamily are composed of a series of triple helical repeats that serve as binding sites for other cytoskeletal proteins. Spectrin also harbors a central SH3 domain and binding sites for Ca^2+^- activated proteins, suggesting that it may play a role in signaling cascades. The actin-binding domain (ABD) is limited to the N-terminal region of βI-spectrin and is composed of two calponin homology domains.

As a first step in characterizing the role of spectrin in dendritic spines, we transfected CA1 pyramidal neurons in hippocampal slice cultures with the ABD of spectrin or closely related proteins, following the approach previously used to study α-actinin [30]. We suggest that βI-spectrin may play both a structural and functional role in dendritic spines through interaction with the actin cytoskeleton and Rac3.

## Methods

### Ethical approval

All experimental protocols that involve tissue collected from animals in this study have been approved by The University of Maryland School of Medicine Institutional Animal Care and Use Committee (Protocol #0609015).

### Neuronal cultures

Organotypic hippocampal slice cultures were prepared with the roller tube method [31]. Briefly, hippocampi were dissected from 5–7 day-old rat pups and cut into 400 µm-thick slices with a chopper, under sterile conditions. Slices were attached to cleaned, poly-lysine–coated coverslips in a mixture of lyophilized chicken plasma (Cocalico, Reamstown, PA) and concentrated fibrin solution (Tisseel, Baxter Health Care) that was then clotted with thrombin. Coverslips containing slices were placed in culture tubes on a slowly rotating roller drum in an incubator. After 4 days *in vitro*, cultures were treated with antimitotics overnight to reduce glial proliferation. Cultures remained in the incubator for 12–16 days before transfection. Cortical neuronal cultures were prepared and cultured from E18 rat neocortex, as described [12].

### ABD and Rac constructs

Plasmids were created using PCR to amplify the cDNAs of the actin-binding domains (ABDs) of dystrophin, utrophin, actinin-2, and βI-spectrin [detailed methods: 32]. The first 942 base pairs of the coding sequence of the ABD of βI-spectrin (NM_000347) was amplified from plasmid B259 [32]. With primers A, 5′ CGTAGAAT TCCTATG-ACATCGGCCACAG 3′ (sense), and B, 5′ TCCCCGCGGCTAAGGTGAG CAGGTCCGA 3′ (antisense). cDNA was cloned into the DsRed2-c1 plasmid vector (BD Biosciences Clontech) via a EcoRI sense site and a SacII antisense site. For dystrophin, a plasmid (pDys246) [33] was used with primers A, 5′ GCGAATTCTATGTTGTTGTGGGAAGAAGTA 3′ (sense), and B, 5′ GCAGGTACCCTATTCAATGCTCACTTGTTG 3′ (antisense), to amplify and clone the first 738 bases of the dystrophin gene (accession no. M18533), containing its ABD. The dystrophin ABD was cloned into the DsRed2-c1 vector via an EcoRI site on the sense primer and a KpnI site on the antisense primer. An utrophin plasmid (pet23Utr261) [34] served as a template to amplify the ABD. The primers A, 5′ TATAAGCTTC-TATGGCCAAGTATGGGAC 3′ (sense) and B, 5′ TGTGGATCCCTAATCTATCGTGACTT-GCTG 3′ (antisense) were used to amplify the first 783 bases of the utrophin gene (accession no. Y12229), which was then cloned into the DsRed2-c1 vector via a HinDIII sense site and a BamHI antisense site. The ABD of α-actinin-2 (CAA32078.1) was cloned into the DsRed2-c1 vector similarly, with the primers. A, 5′ GTAGAATTCTATGAACAGCATGAACCAG 3′ (sense) and B, 5′ TGTGGATCCCTATTTCTCAGCAATTTCCATG 3′ (antisense) were used to amplify the first 671 bases of the α-actinin-2 gene, via an EcoRI sense site and a BamHI antisense site. GFP-actin was from Clontech (Mountain View, CA). cDNAs encoding wild type and dominant negative Rac 1 and 3 were myc- and HA-tagged constructs from the Guthrie cDNA Resource Center (Rollo, MO). Plasmids were introduced into bacteria and amplified using standard protocols. Amplified cDNA was isolated and purified using a HiSpeed Plasmid Maxi Kit (Qiagen; Valencia, CA) following the manufacturer's instructions and prepared for the biolistic transfection of organotypic hippocampal slice cultures.

### Biolistic transfection

Cultures were transfected biolistically with a gene gun (Helios; Bio-Rad, Hercules, CA) as described [35]. Gold pellets (1.0 µm diameter) were coated with spermidine and then placed in a solution of 25 µg/µl DNA, which was precipitated onto the particles by the addition of CaCl_2_ to a final concentration of 500 mM. The coated pellets were attached to the walls of the cartridges with polyvinylpyrrolidone, as described. Prior to transfection, each slice was placed in a small volume of solution containing 40 µM amino-5-phosphonovaleric acid (AP5), 40 µM 6,7-dinitroquinoxaline-2,3-dione (DNQX), and 10 mM Mg^2+^ to block synaptic transmission and reduce excitotoxicity. Cultures were transfected by shooting the gold particles out of the gene gun through a nylon mesh (90 µm^2^ pore size) at a distance of 2 cm from the culture surface at a pressure of 200 psi. After transfection, slices were returned to roller tubes and placed in the incubator for 24–48 hrs before being used for electrophysiology or imaging.

### Electrophysiology

Cultures were placed in a recording chamber and perfused with extracellular saline containing (in mM): 145 NaCl, 10 NaHCO_3_, 2 CaCl_2_, 2 MgCl_2_, and Phenol Red (10 mg/l). Transfected neurons were identified by fluorescence microscopy before recordings were performed. Patch pipettes were filled with the following (in mM): 130 gluconic acid, 10 KCl, 1 MgCl_2_, 2 ATP, 10 HEPES, and 0.1 EGTA, titrated to pH 7.4 with KOH. To ensure that transfected neurons were those being recorded from, the pipette solution also contained Alexa 488 (100 µM). After breakthrough to whole-cell configuration, transfected neurons contained both green Alexa 488 and red DsRed fluorescence, whereas untransfected cells, recorded within the same slice culture, displayed only green fluorescence. Miniature excitatory postsynaptic currents (mEPSCs) were recorded at a holding potential of −70 mV in the presence of tetrodotoxin (1 µm) and bicuculline (40 µm) at room temperature. Recording pipette resistances were between 6.5 and 8 MΩ. Recordings in which the access resistance exceeded 30 MΩ were discarded. Spontaneous mEPSCs were amplified using an Axopatch 200B amplifier and Clampex 9 software (Molecular Devices, Sunnyvale, CA), low-pass filtered at 2 kHz, and digitized at 10 kHz. mEPSCs were identified and characterized using a template-matching algorithm created from the average of 50 events. To be included for analysis, mEPSCs had to have a uniphasic rise time of <6 ms, a decay time constant of <25 ms and a peak amplitude >4 pA. Decay time constants were calculated by fitting each event with a single exponential.

### Microscopy and data analysis

In experiments involving static imaging, spine head width and spine length were measured from confocal micrographs of all morphological spine categories. Confocal images were taken with an upright Zeiss LSM-510 Meta Confocal Microscope System using a water immersion objective (Zeiss; 60X/NA 1.2, C-Apochromat, effective pixel size = 0.017 µm^2^). Fluorescence excitation was achieved using a 25 mW Argon ion laser (Spectraphysics, Mountain View, CA) and illuminating the specimen using the 488 nm and 514 nm lines.

3D image stacks were collected from living cells in unfixed cultures, background fluorescence was subtracted, and images were collapsed into maximal fluorescence 2D projections. Spines were then traced manually and the spine head width or the spine length was computed using the SimplePCI software with the experimenter blinded to the condition. Spine head width was defined as the maximum dimension of the spine head, whereas spine length was defined as the distance from the base of the spine neck to the tip of the spine head. Spine density was calculated by manually counting the number of spines expressed over a 30 µm segment of dendrite. For all measurements of spine density or dimensions, at least 2 well isolated apical dendrites were used per neuron. In experiments involving dynamic imaging, these same analyses were performed on images taken from living cells in unfixed cultures collected using an epifluorescence wide-field microscope (Nikon; 60X/0.8 NA, water immersion objective) and a CCD camera (Hamamatsu Orca ER; effective pixel size = 0.012 µm^2^). Photodamage was minimized by adding Trolox (0.2 mM) to the saline.

For post-hoc analysis of time-lapse image sequences, spine heads were traced manually and the length and width were computed. This method underestimates the morphological dynamics of spines because the measurements are only taken in x and y planes [36]. Images were captured every second for 3 min and binned into 10 s intervals. Morphological dynamics were measured by calculating the coefficient of variation of the spine head diameter within each 10 s interval. Out-of-focus images were removed from the analyses. Fluorescence intensity was calculated after subtracting the average background intensity of two regions of interest surrounding the dendritic spine.

To measure PSD-95 area, slice cultures were co-transfected biolistically with GFP fused to PSD-95 and DsRed fused to the βI-spectrin ABD. PSD-95 - GFP was a gift from Dr. T. A. Blanpied, and was constructed by subcloning PSD-95 at HindIII-EcoR1 into N1 versions of the fusion protein expression constructs. After 24–48 h, slices were placed in a standard recording chamber filled with control saline and wide-field images were taken, as above. After background subtraction, each cluster of PSD-95-GFP was traced manually and an area was calculated using SimplePCI software.

Analyses of dendritic spine dimensions and PSD-95-GFP area were performed by comparing the cumulative probability distributions with the Kolmogorov-Smirnov test. Otherwise, unpaired t-tests were used to establish significance. When comparing more than two groups, ANOVA was used with a Tukey's or Scheffe's post-hoc analysis to establish significance unless otherwise noted. All values are presented as mean values ± standard error of the mean.

### Immunoprecipitation

Cultures of cortical neurons were washed with buffered saline with complete protease inhibitors (Roche Applied Science, Indianapolis, IN), placed in 0.32 M sucrose in the same solution, and scraped. After brief centrifugation, the cellular pellet was mixed with an equal volume of two-fold concentrated RIPA buffer to obtain the following final concentrations of detergents: 1% NP-40, 0.5% deoxycholate, 0.1% SDS. Extracts were triturated in the cold, briefly, and stored on ice. Aliquots (70 µl) of Dynabeads (Invitrogen, Carlsbad, CA), pre-coated with sheep anti-rabbit IgG, in buffered saline were bound with 6 µg affinity-purified rabbit antibodies to βI-spectrin or with purified non-immune rabbit IgG and washed. The bead pellet was mixed with 1 mg of protein of the cellular extract and mixed gently overnight. The immunoprecipitates were collected, washed briefly in buffered saline containing 0.5% Tween, solubilized with SDS-PAGE sample buffer, and subjected to SDS-PAGE, followed by electro-transfer to nitrocellulose. Blots were incubated with antibodies to βI-spectrin, Rac1, or Rac3. Bound antibody was detected with peroxidase-conjugated secondary antibodies (Roche Diagnostics, Indianapolis, IN; Amersham Biosciences, England) and the SuperSignal West Femto Kit (Thermo Scientific, Waltham, MA). Samples of the cell extract were run in parallel, to mark the positions of the proteins of interest. Affinity-purified rabbit antibodies to βI-spectrin have been described [37]. Antibodies to Rac1 were from Santa Cruz Biotechnology (Santa Cruz, CA). Antibodies to Rac3 were the generous gift of Dr. I. De Curtis (San Raffaele Scientific Inst., Milan, Italy).

## Results

### The ABD of βI-spectrin induces actin bundling in CA1 pyramidal neurons

**Figure 1:**
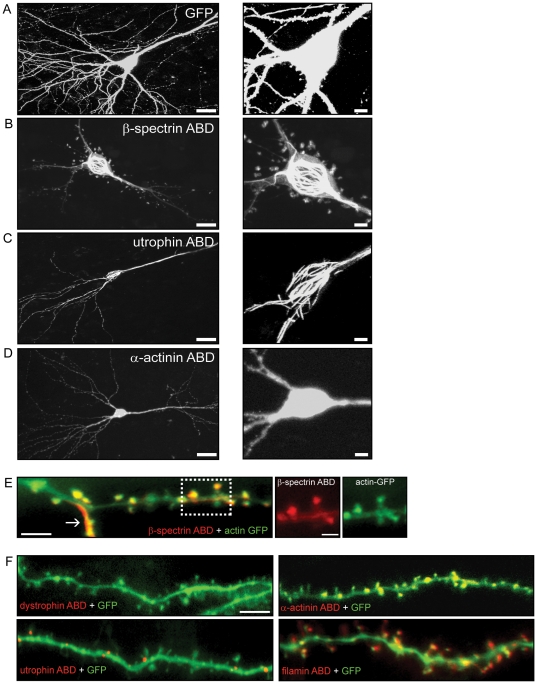
Actin binding domains from various spectrin superfamily proteins differentially bundle actin and localize to spines in CA1 pyramidal neurons. Two-dimensional projections of confocal images of neurons expressing GFP (***A***) or the ABDs of different proteins fused to DsRed (***B–F***). (***B***) Neurons expressing the ABD of βI-spectrin show actin bundling in the cell soma and in dendrites. The ABD of utrophin (***C***) also bundles actin in the soma. Neurons expressing the ABD of actinin (***D***) did not display actin bundling in the soma, dendrites, or spines (Scale right = 25 µm, Scale left = 100 µm). ***E***, Merged image of a dendrite from a CA1 pyramidal neuron co-expressing actin-GFP and the ABD of βI-spectrin fused to DsRed. The arrow indicates a bundled actin filament in the dendrite. Images at the right show the co-localization of actin and the ABD of βI-spectrin in spine heads (scale bar = 1 µM). ***F***, Co-expression of the ABDs of α-actinin, utrophin, dystrophin, or filamin, all fused to DsRed, and GFP in CA1 pyramidal cells demonstrate their differential targeting to dendritic spines (***E***, ***F*** Scale bar = 10 µm).

To investigate the interaction between spectrin family proteins and the actin cytoskeleton, we created constructs of the highly homologous ABDs of βI-spectrin, α-actinin-2, utrophin, dystrophin, and filamin (supporting information Fig. S1), all fused to the fluorophore DsRed, and introduced them biolistically into CA1 pyramidal cells in hippocampal slice cultures. We co-transfected with a GFP plasmid in most experiments to fill the cells and allow visualization of dendrites and dendritic spines (Fig. 1A), and quantified spine shape and size from images of GFP distribution.

The DsRed-tagged βI-spectrin ABD labeled filamentous structures in the cell body and apical and basal dendrites (n = 12)(Fig. 1B). We observed a distribution of the ABD of βI-spectrin that was similar to what has been reported for endogenous spectrin in hippocampal pyramidal neurons [29]. Co-transfection of GFP-tagged actin revealed that these structures were bundles of filamentous actin, which were not seen with expression of actin-GFP alone, suggesting that the ABD of βI-spectrin associates with actin filaments, promoting their formation and inducing bundling. The ABD of βI-spectrin, either alone or together with actin-GFP, also labeled spine heads in apical and basal dendrites (Fig. 1E).

Expression of the ABD of other members of spectrin superfamily tagged with DsRed gave different results. Expression of the ABD of utrophin resulted in actin bundling in the neuronal cell bodies, as well as apical and basal dendrites (n = 12)(Fig. 1C), but minimal labeling of dendritic spines (Fig. 1F). Expression of the ABDs of actinin-2 (n = 6) and filamin (n = 7), in contrast, produced diffuse labeling of the soma and principal dendrites (Fig. 1D), without actin bundling, as well as prominent labeling of dendritic spines (Fig. 1F). The ABD of dystrophin (n = 12) produced no actin bundling in the cell bodies or dendrites and did not label spines (Fig. 1F). Co-transfection of GFP with the utrophin and dystrophin constructs revealed that spines were present, but unlabeled by their ABDs. We observed that the expression level for all of the ABD constructs were similar based on the distribution and fluorescence intensity of each ABD. These results demonstrate that the ABDs of spectrin superfamily members caused distinct effects on actin filaments and were targeted differentially to dendritic spines. Although these ABDs are highly homologous, their effects are therefore likely to be mediated by more than mere binding to actin.

### The ABD of βI-spectrin enlarges dendritic spine heads

**Figure 2:**
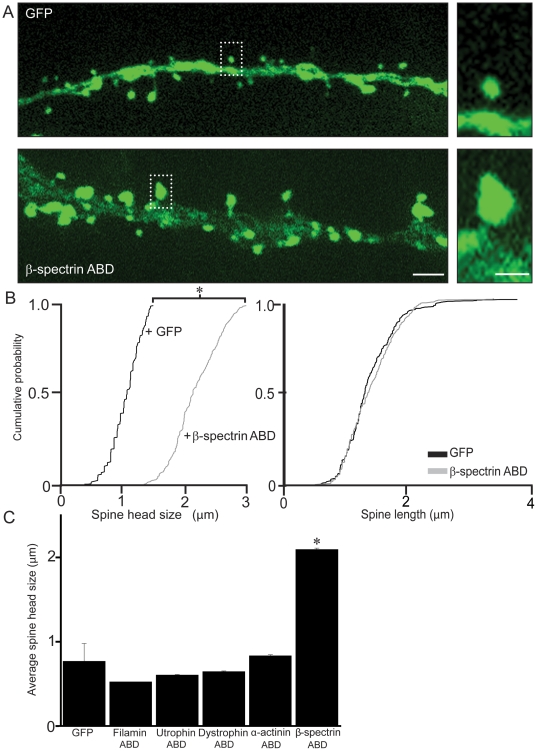
The ABD of βI-spectrin specifically enlarges dendritic spine heads. ***A***, Images of GFP fluorescence in cells co-transfected with GFP and the ABD of βI-spectrin fused to DsRed, or with GFP alone, illustrate the substantial increase in spine head diameter seen when the ABD of βI-spectrin is overexpressed. Scale = 4 µm (left) and 2 µm (right). ***B***, Cumulative probability plots demonstrate the significant increase in spine head diameter in cells expressing ABD of βI-spectrin when compared to cells transfected with GFP alone, and the lack of a significant effect on spine length (n = 500 spines/5 neurons/group; * = p<0.05, K-S test). ***C***, When compared to the ABD of other spectrin superfamily proteins, only the spines expressing the ABD of βI-spectrin were significantly enlarged (n = 5 neurons/construct; * = p<0.01, ANOVA with Tukey's test).

**Figure 3:**
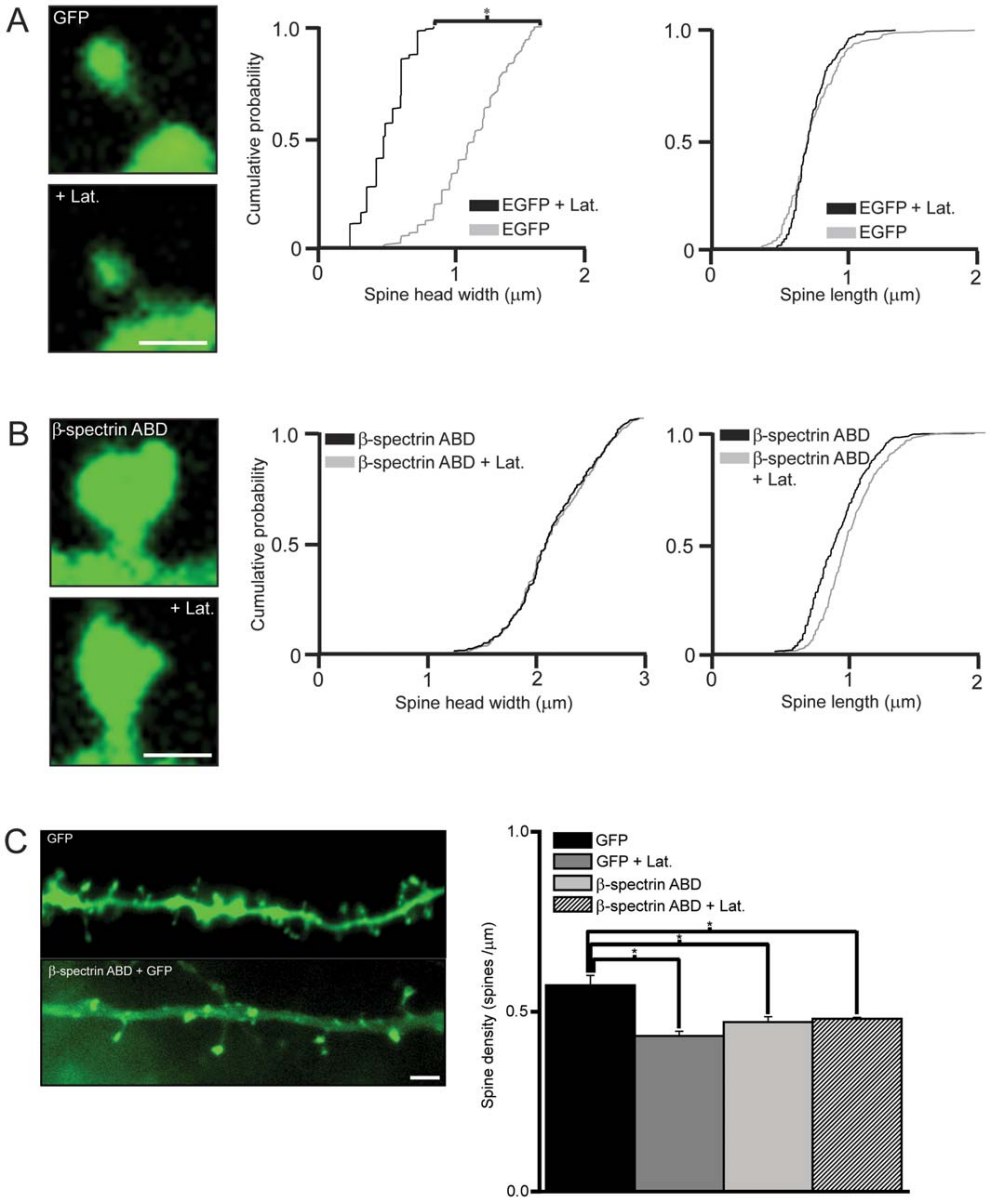
The ABD of βI-spectrin prevents actin depolymerization. ***A***, After application of latrunculin (5 µm) for 5 hr, the diameter of the spine head in cells expressing GFP alone decreased significantly (n = 500 spines/5 neurons/group; p<0.05, K-S test), whereas spine length was not changed. ***B***, Latrunculin treatment produced no significant decrease in spine head diameter or spine length in cells co-expressing the ABD of βI-spectrin fused to DsRed and GFP, in contrast. Scale bar = 2 µm for **A** and **B**. ***C***, Application of latrunculin-B resulted in a significant decrease in spine density in spines expressing GFP alone (n = 5 neurons/group; p<0.01 ANOVA with Tukey's test), but not in spines expressing the ABD of βI-spectrin. Spine density was significantly lower in cells expressing the β1 spectrin ABD, as compared to cells expressing GFP alone. Scale bar = 6 µm.

Endogenous βI-spectrin is expressed at high levels in the heads of postsynaptic spine [29]. Analysis of confocal images of GFP-filled dendritic spines revealed that the heads of dendritic spines co-expressing the ABD of βI-spectrin were more than twice as large as spines expressing GFP alone (n = 500 spines/5 neurons/group; p<0.05, K-S test)(Fig. 2). Spine length was not significantly different between the two groups, however (n = 500 spines/5 neurons/group, p>0.05, K-S test)(Fig. 2B). Neurons co-expressing the ABD of βI-spectrin and GFP also displayed a small (∼20%), but significant, decrease in average spine density as compared to GFP transfected neurons (n = 5 neurons/group p<0.01, ANOVA and Tukey's test)(Fig. 3C).

Only the ABD of βI-spectrin affected dendritic spines. Dendritic spine heads in cells expressing GFP plus the ABDs of α-actinin-2, dystrophin, utrophin and filamin were not significantly larger than spines in cells expressing GFP alone (n = 5 neurons/group p>0.05, ANOVA and Tukey's test)(Fig. 2C). Because all of these constructs bind actin, some other action of the βI-spectrin ABD must be responsible for the enlargement of the dendritic spine head.

### The ABD of βI-spectrin stabilizes actin filaments in dendritic spines

Differences in the sizes of spine heads correlate with different states of actin polymerization [38]. We further probed the activity of the ABD of βI-spectrin on actin by testing its ability to stabilize actin filaments in spines in the presence of latrunculin-B, which depolymerizes F-actin by preventing addition of G-actin monomers [15], [39]. F-actin in spines is significantly depolymerized after 1 hour of latrunculin treatment, with complete depolymerization requiring 4–8 h. This is accompanied by the collapse of the dendritic spine head, known as “deflation” [15], [21], [39]. In agreement with these results, we found that application of latrunculin-B (6 µM) for 5 h to slice cultures caused a 50% decrease in spine head diameter in cells transfected with GFP alone (GFP+latrunculin = 0.4 µm±0.1 µm vs. GFP alone = 0.8 µm±0.1 µm; n = 500 spines/5 neurons/group; p<0.05, K-S test)(Fig. 3A). Spine length was not affected significantly, however (1.2 µm±0.06 µm control vs. 1.2 µm±0.04 µm latrunculin; n = 500 spines/5 neurons/group; p>0.05, K-S test). In contrast, application of latrunculin to cells transfected with the ABD of βI-spectrin had no significant effect on either spine head diameter (ABD of βI-spectrin+latrunculin = 2.0 µm±0.20 µm vs. ABD of βI-spectrin = 2.1 µm±0.2 µm; n = 500 spines/5 neurons/group; p>0.05, K-S test) or spine length (1.19 µm±0.04 µm control vs. 1.2 µm±0.06 µm latrunculin; n = 500 spines/5 neurons/group; p>0.05, K-S test)(Fig. 3B). Treatment with latrunculin-B also resulted in a significant decrease in overall spine density in GFP expressing neurons (n = 5 neurons/group, p<0.01, ANOVA and Tukey's test), but not in neurons expressing the ABD of βI-spectrin (n = 5 neurons/group, p>0.05, ANOVA and Tukey's test)(Fig. 3C). Actin in spines expressing the βI-spectrin ABD was thus resistant to the depolymerizing actions of prolonged latrunculin treatment.

**Figure 4:**
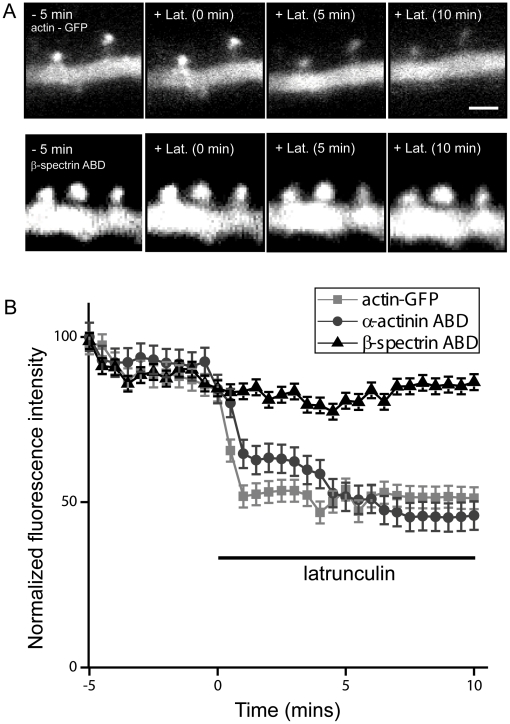
The ABD of βI-spectrin prevents acute actin depolymerization. ***A***, Images of GFP fluorescence in spines from CA1 pyramidal neurons expressing actin-GFP alone (upper row), or co-expressing actin-GFP together with the ABD of βI-spectrin (lower row) before and after the application of 5 µm latrunculin-B. Note that fluorescence decreased within 5 minutes of latrunculin treatment in cells expressing GFP, but not in cells co-expressing the spectrin ABD. Scale bar = 2 µm. ***B***, Normalized fluorescence emission of dendritic spines was imaged every 30 seconds before and after the addition of latrunculin-B. Fluorescence decreased rapidly (<3 min) in spines expressing actin-GFP alone and in spines in which the ABD of α-actinin was co-expressed, but not in spines co-expressing the ABD of βI-spectrin and actin-GFP (n = 5 neurons/construct; p<0.001, repeated measures ANOVA).

We next tested the effects of acute application of latrunculin-B in cells transfected with actin-GFP alone or with the βI-spectrin ABD (Fig. 4). Depolymerization of actin with latrunculin reduces the intensity of actin-GFP fluorescence in the spine head as the actin monomers that cannot polymerize diffuse out of the spine [40]. We found that acute addition of latrunculin-B (5 µm) induced a rapid (<3 min) decrease in actin-GFP intensity in control cells, as previously reported. Latrunculin application had a significantly smaller effect on the intensity of actin-GFP in spines of cells co-transfected with the ABD of βI-spectrin (n = 5 neurons/group, p<0.001, repeated measures ANOVA). This effect was specific for the ABD of βI-spectrin, because latrunculin produced a strong decrease in the fluorescence intensity of spines co-transfected with the ABD of α-actinin-2 and actin-GFP, comparable to its effects on cells expressing actin-GFP alone. We conclude that the presence of the ABD of βI-spectrin in spines stabilizes F-actin and reduces its rate of depolymerization.

### The ABD of βI-spectrin decreases the morphological plasticity of dendritic spines

**Figure 5:**
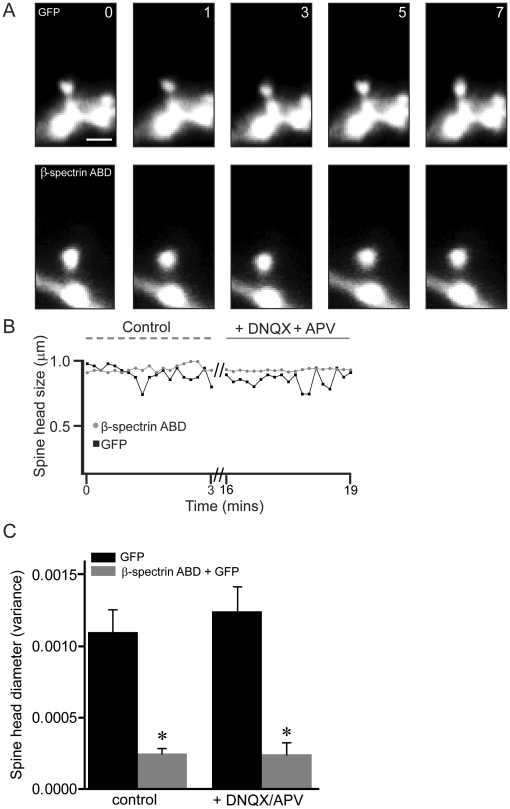
The ABD of βI-spectrin decreases the morphological plasticity of dendritic spines. ***A***, Time-lapse images in control saline of GFP fluorescence in a single spine from a CA1 pyramidal neuron expressing either GFP alone (upper row) or GFP together with the ABD of βI-spectrin (lower row). Rapid, ongoing changes in the shape of the spine head occurred in spines expressing GFP alone, but not in spines expressing both GFP and the ABD of βI-spectrin. Scale bar = 1 µm; time in sec. ***B***, Changes in spine head diameter in the spines from **A** were plotted over time. Spines expressing GFP exhibited more spontaneous morphing, compared to A spine expressing the ABD of βI-spectrin, regardless of whether NMDARs and AMPARs were active. ***C***, Group data showing the variance of spine head diameter in neurons expressing GFP alone or the ABD of βI-spectrin in the two conditions. Spines expressing the ABD of βI-spectrin displayed significantly less variance in the shape of their heads than did spines expressing GFP alone (n = 5 neurons/group; * = p<0.001, Kruskal-Wallis test). There was no significant effect of blocking NMDARs and AMPARs on spontaneous morphing in any condition.

Dynamic morphological changes in the shape of dendritic spine heads (“morphing”) occur on the order of minutes or seconds and are dependent upon actin polymerization and depolymerization in the spine head [10], [40]. We therefore tested if decreases in actin depolymerization resulting from the expression of the ABD of βI-spectrin would decrease dendritic spine motility. We used live-cell imaging to compare the spontaneous, constitutive morphing of dendritic spines in neurons transfected with GFP alone or GFP together with the ABD of βI-spectrin (Fig. 5). Because the activity of both AMPARs and NMDARs may affect spine morphing [10], we also tested the effects of applying saturating concentrations of DNQX (40 µm) and AP5 (40 µm). Using GFP emission, we measured the variance of spine head diameter over a period of 10 min to quantify spine head dynamics and to control for differences in spine head size induced by the constructs. Spines expressing GFP alone exhibited significantly more spontaneous morphing than spines expressing the ABD of βI-spectrin (p<0.001, Kruskal-Wallis test), and neither were affected by DNQX and AP5 (Fig. 5C). We conclude that stabilization of actin filaments by the ABD of βI-spectrin attenuates the structural dynamics of dendritic spines.

### Functional consequences of expressing the ABD of βI-spectrin

**Figure 6:**
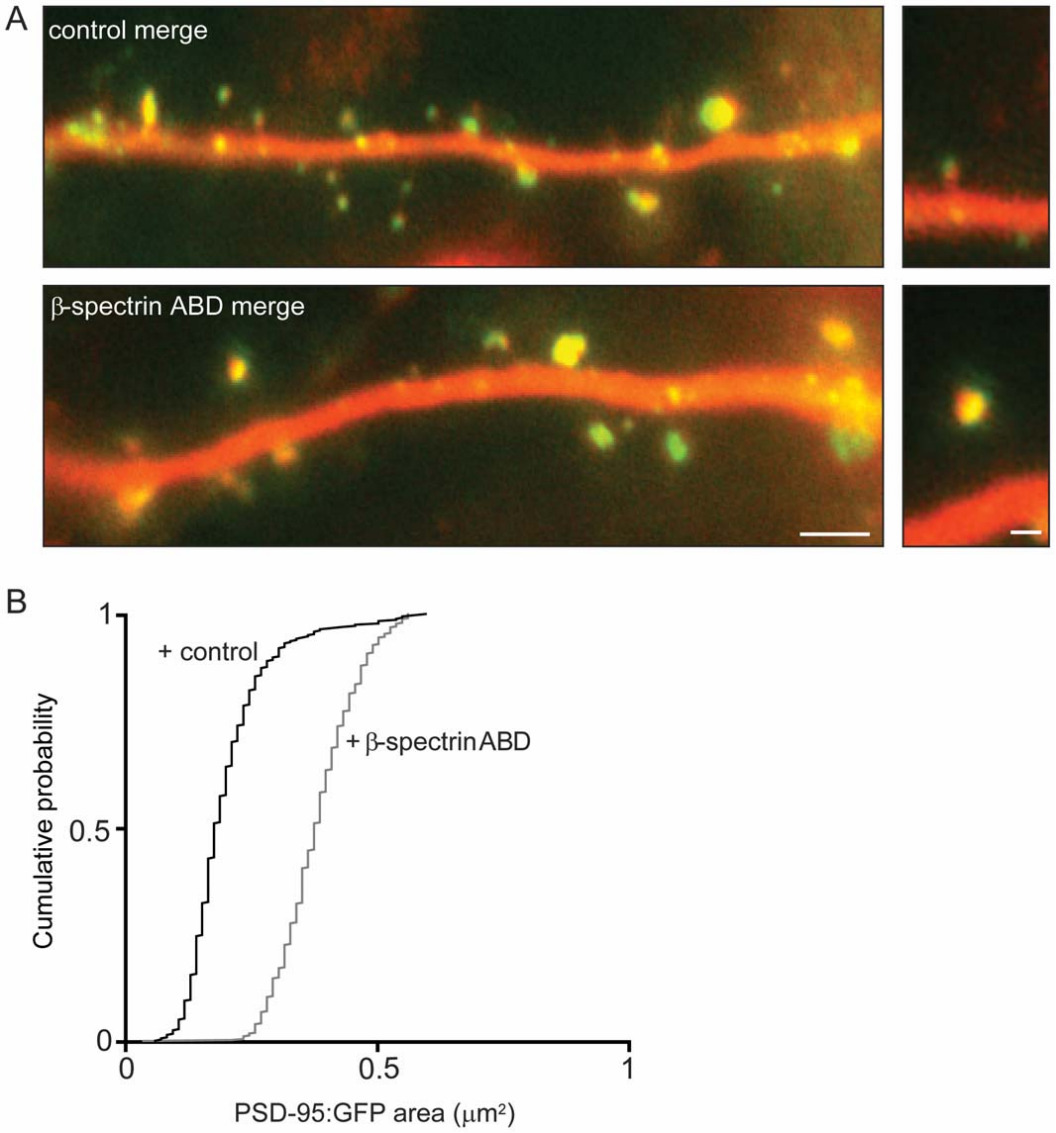
Expression of the ABD of βI-spectrin increases the size of the PSD. ***A***, Merged images from CA1 pyramidal neurons in hippocampal slice cultures co-transfected with PSD-95 fused to GFP (green) and mCherry (red), with or without an unlabelled βI-spectrin ABD construct. The areas of the clusters of PSD-95 in the heads of spines expressing the ABD of βI-spectrin were larger than in cells expressing PSD-95 alone. Scale bar = 4 µm (left), 1 µm (right). ***B***, Cumulative probability plot showing that the area of the PSD was significantly larger in spines expressing PSD-95-GFP together with the ABD of βI-spectrin than in spines expressing PSD-95-GFP alone (n = 400 spines/4 neurons/condition; p<0.05, K-S test).

We next asked if there were functional correlates of the morphological changes induced by the ABD of spectrin. The postsynaptic density (PSD) is a specialized structure at the tip of the dendritic spine head comprised of densely packed glutamate receptors and signaling proteins [41], [42] and its size is positively correlated with synaptic strength [2]. A principal constituent of the PSD is the scaffolding protein, PSD-95 [43]. As a first test of the possibility that the enlargement of spine heads induced by the ABD of βI-spectrin (lacking the DsRed tag) would be associated with an increase in synaptic strength, we co-expressed it with GFP-tagged PSD-95 and cytosolic mCherry (Fig. 6A). We observed a significant increase in the area of PSD-95-GFP in dendritic spines co-expressing the ABD of βI-spectrin, as compared to cells expressing PSD-95-GFP alone (mean area = 0.20 µm^2^±0.02 µm^2^ in controls vs. 0.38 µm^2^±0.01 µm^2^ with the ABD of βI-spectrin; n = 365 spines/4 neurons/group; p<0.05, K-S test)(Fig. 6B). In agreement with previous reports [44] we observed a linear relationship between spine head size and PSD-95 area under both conditions (r = 0.83 for control and 0.79 for the ABD of βI-spectrin). We conclude that overexpression of the ABD of βI-spectrin is associated with an increase in the size of the PSD.

**Figure 7:**
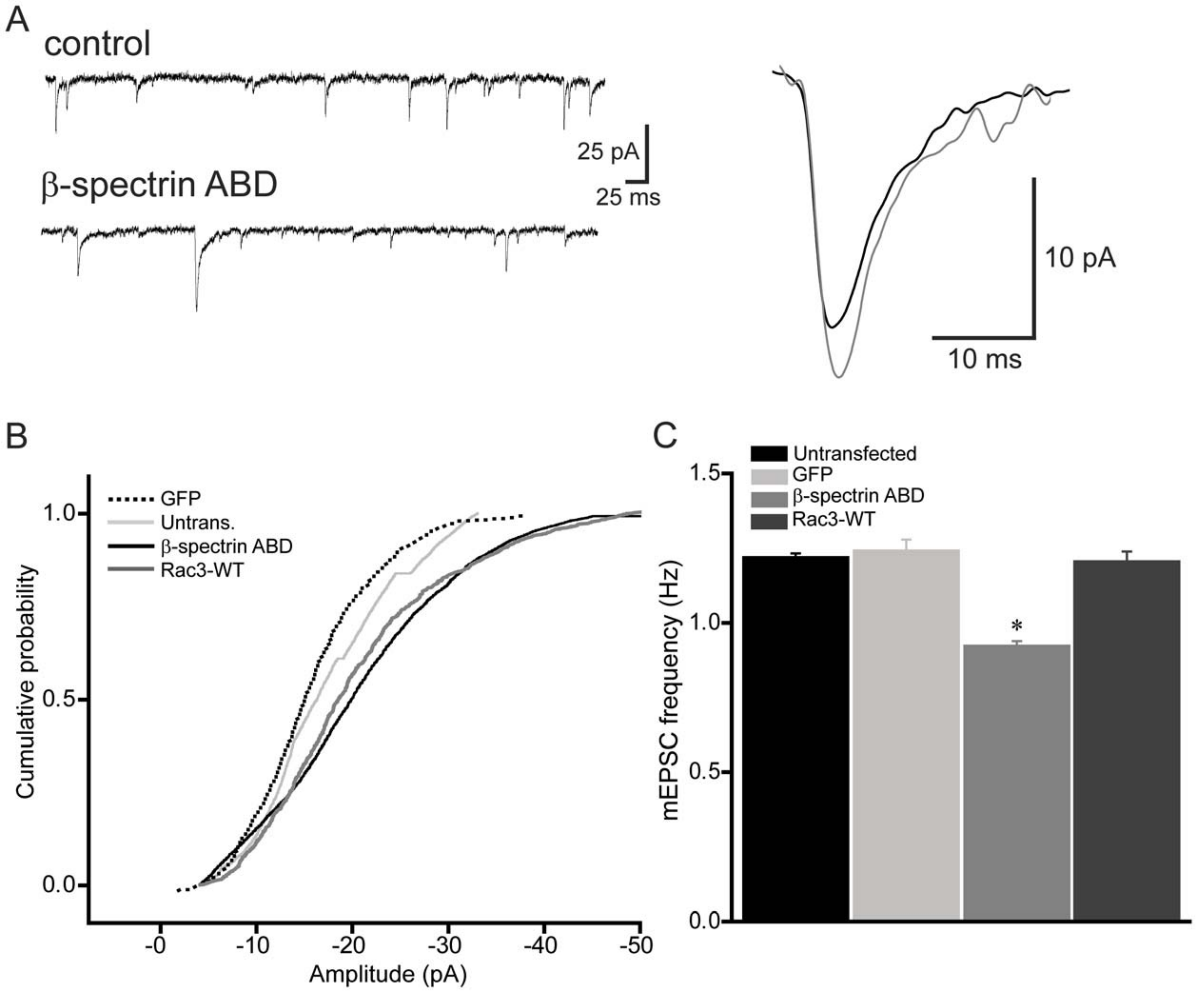
The ABD of βI-spectrin enhances single spine AMPAR-mediated synaptic responses. ***A***, mEPSCs from an untransfected control cell (upper trace) and a cell transfected with the ABD of βI-spectrin (lower trace) were recorded at −70 mV in the presence of TTX (1 µm). Average sized single mEPSCs recorded from the untransfected cell (black) and transfected cell (gray) are shown superimposed at right. ***B***, Group data showing that neurons expressing the ABD of βI-spectrin had significantly greater mEPSC amplitudes, compared to untransfected controls or cells expressing GFP alone, suggesting an increase in AMPARs at these synapses (n = 7 neurons/group; p<0.05, K-S test). The increase in mEPSC amplitude produced by the spectrin ABD was mimicked in cells expressing CA Rac3, in which mEPSC amplitude was also larger than in controls or GFP expressing cells (p<0.05, K-S test). ***C***, The frequency of mEPSCs in transfected neurons decreased significantly, suggesting fewer functional synapses (n = 7 neurons/group;* = p<0.05, t-test).

It has been demonstrated that the number of AMPARs is proportional to PSD area [2], [45], [46]. We therefore asked whether the increase in PSD size produced by the ABD of βI-spectrin would be accompanied by an increase in the number of synaptic AMPARs in the spine head. To answer this question, we transfected CA1 pyramidal cells in hippocampal slice cultures with the ABD of βI-spectrin tagged with DsRed and performed whole-cell voltage-clamp recordings of spontaneous miniature excitatory postsynaptic currents (mEPSCs) on transfected and untransfected neurons in the presence of TTX (1 µm). Alexa 488 was included in the pipette solution in order to ensure that the recorded neurons were truly those expressing the spectrin construct, as apparent by green and red fluorescence. Mean mEPSC amplitudes were significantly larger in cells transfected with the ABD of βI-spectrin compared to untransfected cells in the same cultures, consistent with an increase in the number of synaptic AMPARs (mean amplitude = 20.8±1.4 pA for cells expressing the ABD of βI-spectrin vs. 17.1±1.1 pA in untransfected cells; n = 7 neurons/group; p<0.05, K-S test)(Fig. 7A,B). The frequency of mEPSCs was decreased ∼20% after transfection with the ABD of βI-spectrin, compared to untransfected neurons recorded from the same slice (p<0.05, t-test)(Fig. 7C), consistent with the decrease in spine density described above. To test for non-specific effects of biolistic transfection on neuronal function, we also compared mEPSC amplitudes in neurons transfected with GFP alone and untransfected cells and observed no significant difference (mean amplitude = 17.1±0.5 pA in untransfected cells vs. 15.7±0.4 pA in cells expressing GFP; n = 7 neurons/group, p>0.05, unpaired t-test). The presence of the ABD of βI-spectrin in dendritic spines thus increased the sizes of the PSD and the spine head, as well as synaptic strength.

### The activity of the ABD of βI-spectrin is mediated by Rac3

Do the effects of the βI-spectrin ABD on spines result directly from its interaction with actin or do they result because of recruitment of some other molecule to the spine head? Our observation that only the ABD of βI-spectrin localizes to spines and caused their enlargement, although all of them bind to actin (Fig. 2), favors the later hypothesis. Small GTPases are important signaling molecules involved in the regulation of dendritic spine morphological dynamics [47]. One small GTPase, Rac1, modulates dendritic spine morphology [48] and function [49]. We therefore tested the role of rac proteins in the morphological changes induced by the ABD of βI-spectrin.

**Figure 8:**
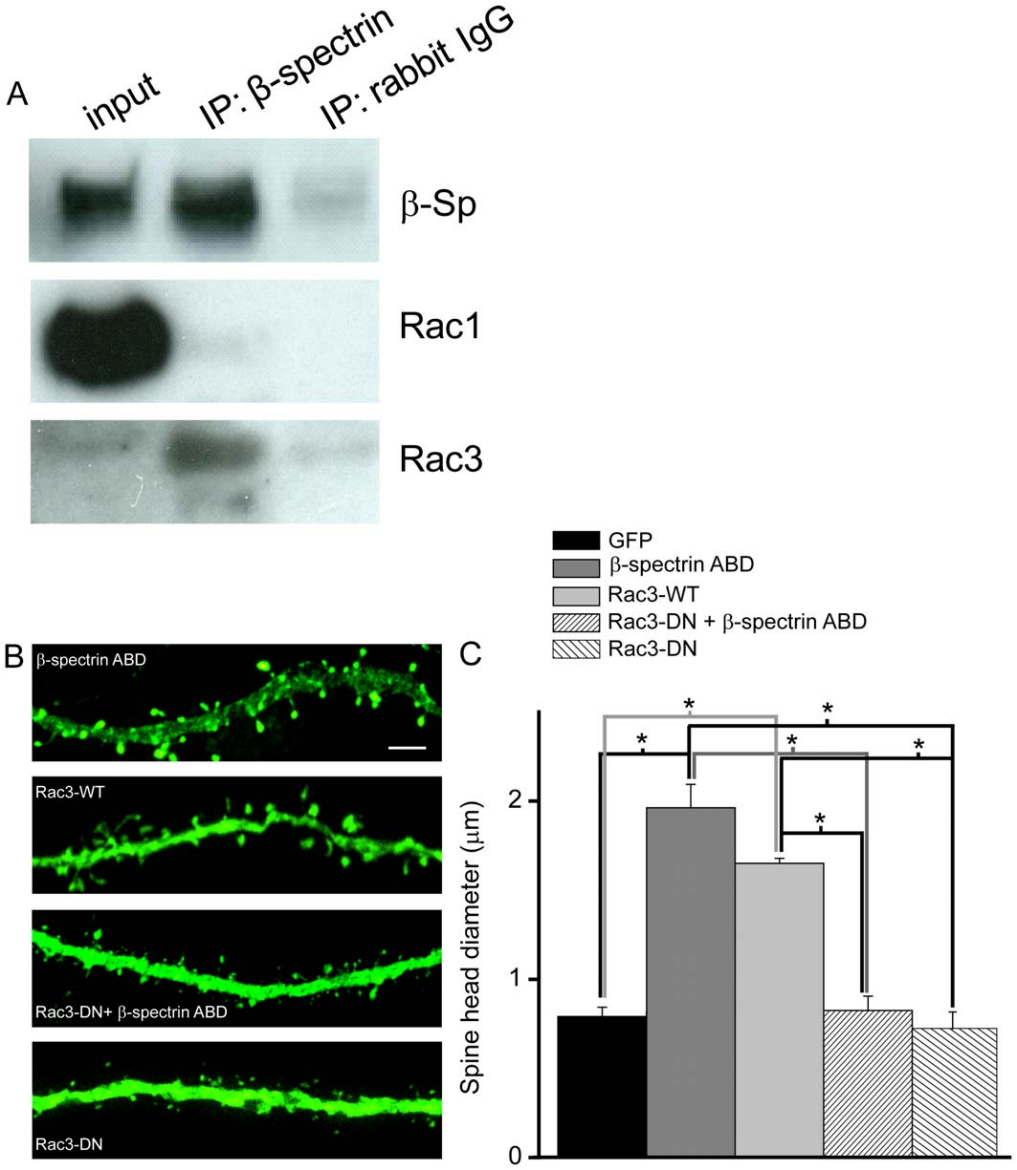
Rac3 is immunoprecipitated with the ABD of βI-spectrin and might mediate its effects on dendritic spines. ***A***, Rac3 is specifically enriched in immunoprecipitates of detergent extracts of cortical cell cultures generated with antibodies to βI-spectrin. The input sample (lane 1) contained easily detected amounts of βI-spectrin and Rac1 but relatively little Rac3. Immunoprecipitation with an anti-βI-spectrin antibody (lane 2) specifically concentrated βI-spectrin and Rac3, but did not precipitate Rac1. Neither GTPase was detected after immunoprecipitation with a non-immune rabbit IgG (lane 3). ***B***, GFP images of CA1 pyramidal cell dendrites co-expressing GFP and the ABD of βI-spectrin either with or without dominant negative (DN) Rac3, and co-expressing GFP with wild type Rac3. Scale bar = 10 µm. ***C***, Pooled data illustrate the effects on the mean spine head diameter, calculated from GFP images (n = 100–300 spines/neuron, 2–5 neurons/group, p<0.001, ANOVA with Sheffe's test). Spines in cells expressing the ABD of βI-spectrin had larger heads than cells expressing GFP alone, but not when co-expressing dominant negative Rac3. Expression of wild-type Rac3 resulted in spines with large heads, thus mimicking the effects of the ABD of βI-spectrin.

Two Rac GTPases are expressed in hippocampal and neocortical pyramidal cells, Rac1 and Rac3 [50], [51]. We first immunoprecipitated endogenous βI-spectrin from cultured cortical neurons and screened for the presence of Rac3 and Rac1. Surprisingly, Rac3 was enriched in immunoprecipitates of βI-spectrin, but Rac1 was absent (3 replicates)(Fig. 8A). We tested the importance of this interaction by co-expressing a dominant negative Rac3 construct together with the ABD of βI-spectrin. Under these conditions, spine head diameters were significantly smaller than in cells expressing the βI-spectrin ABD alone (Fig. 8B,C). Spine head diameters in cells co-expressing the βI-spectrin ABD and dominant negative Rac3 or Rac3-DN alone were not significantly different than in cells expressing only GFP, suggesting that Rac3 signaling is required for the effects of the βI-spectrin ABD on spines. If so, then this effect should be mimicked by over-expressing wild-type Rac3. Indeed, the size of dendritic spine heads in CA1 pyramidal cells transfected with Rac3 was comparable to spines in cells expressing the ABD of βI-spectrin. Overexpression of Rac3 also resulted in an increase in mEPSC amplitude. The mean amplitude of mEPSCs recorded from neurons expressing Rac3 was not significantly different from those expressing the ABD of βI-spectrin and both were larger than for mEPSCs in untransfected or GFP-transfected cells (Fig. 7B). We conclude that Rac3 can alter dendritic spine structure and function in hippocampal pyramidal cells.

## Discussion

Actin is highly concentrated in dendritic spines and determines their morphology [52]–[54]. The actin cytoskeleton provides a scaffold to which a variety of transmembrane proteins and their intracellular signaling partners are anchored, often indirectly via actin binding proteins. The spectrin superfamily of proteins can form complexes via their actin binding domains, which interact with a number of other proteins, such as RACK-1, protein 4.1 and dynactin [25], [55], [56]. βI-spectrin has been immunocytochemically detected in dendritic spines, but its functions there remain unknown.

In this study, we expressed the actin binding domain of βI-spectrin and related spectrin superfamily proteins, an approach used successfully as a first step in revealing the function of the full-length proteins, such as α-actinin [30]. Our assumption is that the exogenous ABD construct will mimic the function of the N-terminal ABD of the full length protein. We cannot exclude, however, that overexpression of the ABD of βI-spectrin may negatively affect endogenous spectrin or induce other downstream compensatory effects [30]. Indeed, a full characterization of spectrin requires complimentary approaches, such as knock-down of endogenous spectrin and overexpression of the full length protein, that are beyond the scope of the current study. Nevertheless, spectrin is a prominent substrate of the Ca^2+^-activated protease calpain and activation of calpain may result in the creation of fragments not unlike those we have expressed.

### Interactions between βI-spectrin and actin

Although all of the proteins of the spectrin superfamily and related proteins share highly homologous ABDs, they are all unique at many sites (Fig. s1). Indeed, we found a surprising heterogeneity in the way these ABDs were targeted in CA1 pyramidal cells. The ABDs of βI-spectrin and utrophin, but not dystrophin, colocalized with GFP-tagged actin and bundled actin filaments in the cell body and proximal dendrites. The ABDs of βI-spectrin, α-actinin-2 and filamin, but not utrophin and dystrophin, were concentrated with actin in the heads of dendritic spines. Our results with the ABD of α-actinin-2 are in contrast to those of Nakagawa et al. [30], who reported that the ABD of α-actinin-2 did not localize to spines, although comparable regions of the protein were used in both studies. Even more unique to the ABD of βI-spectrin was its ability to enlarge the spine head. None of the other ABDs we assayed produced this effect. Nakagawa et al. [30] observed that overexpression of full length α-actinin-2 elongated dendritic spines and that this required its ABD. These results suggests that different actin-binding proteins from the spectrin superfamily can exert differential structural effects on dendritic spines, but only the ABD of βI-spectrin can enlarge postsynaptic spine heads.

The ABD of βI-spectrin also affected the stability of actin filaments within the spine. In control cells, both acute (minutes) and chronic (5 hr) application of latrunculin-B, an inhibitor of actin polymerization, reduced the dimensions of spine heads and caused a loss of GFP-actin from spines, as reported previously [40]. In cells expressing the ABD of βI-spectrin, in contrast, both the latrunculin-induced loss of actin-GFP and deflation of the spine head were inhibited. Thus, the ABD of βI-spectrin reduces the rate at which actin filaments are depolymerized, even when the addition of new actin monomers has been blocked by latrunculin-B. This effect was also specific, as the ABD of α-actinin-2 did not stabilize actin-GFP in spines. These data suggest that some of the effects of expressing these constructs result not from their ability to bind to actin, but perhaps because they recruit other signaling molecules differentially.

Consistent with its ability to stabilize actin filaments, the ABD of βI-spectrin significantly inhibited the constitutive morphing of dendritic spines. Prevention of actin polymerization with latrunculin-B or cytochalasin-D has been reported previously to eliminate dynamic changes in spine head shape [10]. We suggest that endogenous βI-spectrin in spines maintains the spine head in a large and stable state, in part by stabilizing filamentous actin, and that decreases in βI-spectrin might facilitate actin reorganization and promote dynamic changes in spine size and shape.

### Functional consequences of expressing the ABD of βI-spectrin

The turnover of F-actin in dendritic spines is necessary for postsynaptic reorganization and the translocation of proteins into and out of the synaptic plasma membrane [14], [58]. This activity is driven by rapid actin polymerization and depolymerization [59]. For example, inhibition of actin polymerization by latrunculin-A or ADF/cofilin increases the relative rate of AMPAR endocytosis and partially inhibits late-phase LTP [16], [22]. Conversely, the activity of glutamate receptors can affect actin turnover [60] by modulating actin polymerization or depolymerization. Indeed, another spectrin superfamily protein, α-actinin-2, regulates not only the morphology of dendritic spines [57], but also the trafficking of AMPARs [61] and the Ca^2+^-dependent inactivation of NMDARs [62], [63].

Larger dendritic spines contain more AMPARs and generate larger responses to glutamate [2], [46]. We observed an increase in the size of dendritic spine heads after expressing the ABD of βI-spectrin, as well as an increase in the area occupied by PSD-95 in the spine head. Stabilization of F-actin by the βI-spectrin ABD, which increases the size of spine heads, is likely to account for the increase in PSD size [58]. The association of PSD-95 with F-actin is required to anchor AMPARs at postsynaptic sites [64]. We observed that the amplitudes of AMPAR-mediated mEPSCs were significantly larger in cells expressing the ABD of βI-spectrin. The doubling in PSD-95 area and spine head diameter observed in cells expressing the βI-spectrin ABD was associated with only a ∼40% increase in mean mEPSC amplitude, however, indicating that a strict correlation between postsynaptic density size and AMPA receptor number [e.g. 46] is not obligatory under all conditions. Nevertheless, our data represent the first evidence that the actin-binding activity of proteins of the spectrin superfamily can affect postsynaptic sensitivity to released neurotransmitter.

### Rac3 and the ABD of βI-spectrin

How might endogenous βI-spectrin regulate synaptic function? In addition to its ABD, βI-spectrin contains binding sites for calmodulin, protein 4.1, ankyrin, and other proteins [25], [65], [66]. Protein 4.1 promotes the association of the ABD of spectrin with F-actin [67], [68]. The neuronal splice form, 4.1N, interacts with AMPA-type GluR1 subunits and promotes their surface expression [69]. Recruitment of protein 4.1 by the βI-spectrin ABD may therefore promote the insertion of AMPARs into the postsynaptic membrane.

Small GTPases such as Rho, Rac and cdc42, promote morphological plasticity by regulating dynamic changes in the actin cytoskeleton. For example, constitutively active Rac1 disorganizes spines [70], whereas dominant-negative Rac1 disrupts spines [47], [70]. Rac1 activity increases following activation of synaptic NMDARs and promotes the subsequent enlargement of dendritic spines [49]. Karilin-7, a guanine nucleotide exchange factor specific for Rac1, is targeted to dendritic spines, where it enlarges spine heads [71] and potentiates AMPAR-mediated synaptic activity [49]. Less is known about Rac3, although it is highly homologous to Rac1 and is expressed in neurons [51], particularly in the CA1 and CA3 region of the hippocampus during development [50]. Rac3 promotes neurite extension in cultured hippocampal neurons [72] and induces effects on cell differentiation and morphology in neuronal cell lines that are distinct from those caused by Rac1 [73]. Mice in which Rac3 is genetically deleted are viable, learn a hippocampal dependent task normally, and have normal dendritic spine densities, although this may be due to compensation by Rac1 [74], [75].

We observed that Rac3 co-immunoprecipitates with spectrin, but not Rac1. Overexpression of wild-type Rac3 increased the sizes of spine heads as effectively as overexpression of the ABD of βI-spectrin. In contrast, co-expression of a dominant-negative form of Rac3 with the ABD of βI-spectrin with inhibited its ability to enhance spine head size. Finally, neurons that overexpress Rac3 had increased mEPSC amplitudes. We therefore suggest that Rac3 activation is promoted in part by the ABD of βI-spectrin and may mediate some of the effects of endogenous spectrin on postsynaptic structure and function.

How might Rac3 mediate its effects? Rac3 associates with neurabin and this association is required for its GTPase activity [72]. Furthermore, neurabin binds to actin [76] and this binding is required for its localization in dendritic spines [39]. Overexpression of neurabin increases spine head size [77] and AMPAR expression and triggers actin bundling [78]. It is thus possible that the presence of high levels of βI-spectrin ABD in the spine head leads to spine enlargement and synapse strengthening by recruiting Rac3 and neurabin. Alternatively, neurabin may exert its effects on dendritic spines [39] by binding to actin and, like βI-spectrin, recruiting Rac3. It should be noted that the dominant negative Rac3 construct did not *reduce* dendritic spine size compared to control cells. Similarly, genetic deletion of Rac3 does not affect dendritic spine density in hippocampal cell cultures [75], although spine size was not quantified. This suggest that the interaction between endogenous βI-spectrin and Rac3 is not necessary to *maintain* spine head size. This possibility could be tested in future studies in which expression of endogenous βI-spectrin is reduced by targeted siRNA.

Our results suggest the testable hypothesis that the ABD of endogenous βI-spectrin and Rac3 can interact in a complex that promotes both activation of Rac3 and polymerization of actin. Changes in the expression of βI-spectrin might thus affect the dynamic state of the actin cytoskeleton in spines, thereby influencing AMPAR trafficking and the balance between synapse plasticity and stability.

## Supporting Information

Figure S1 **Sequence alignment of spectrin family ABDs.** Although the sequences are identical to β1-spectrin at many residues (indicated by *), and very similar (indicated by :) or homologous at others (indicated by.), there are many differences. Although all share the ability to bind and bundle actin, these regions of difference are presumably responsible for the distinct activities observed in the various experiments.
(TIF)
