# Supplementary figures and images for: The Actin Binding Domain of βI-Spectrin Regulates the Morphological and Functional Dynamics of Dendritic Spines
Source: PLoS One. 2011 Jan 31;6(1):e16197. doi: 10.1371/journal.pone.0016197 (PMC3031527; doi:10.1371/journal.pone.0016197)

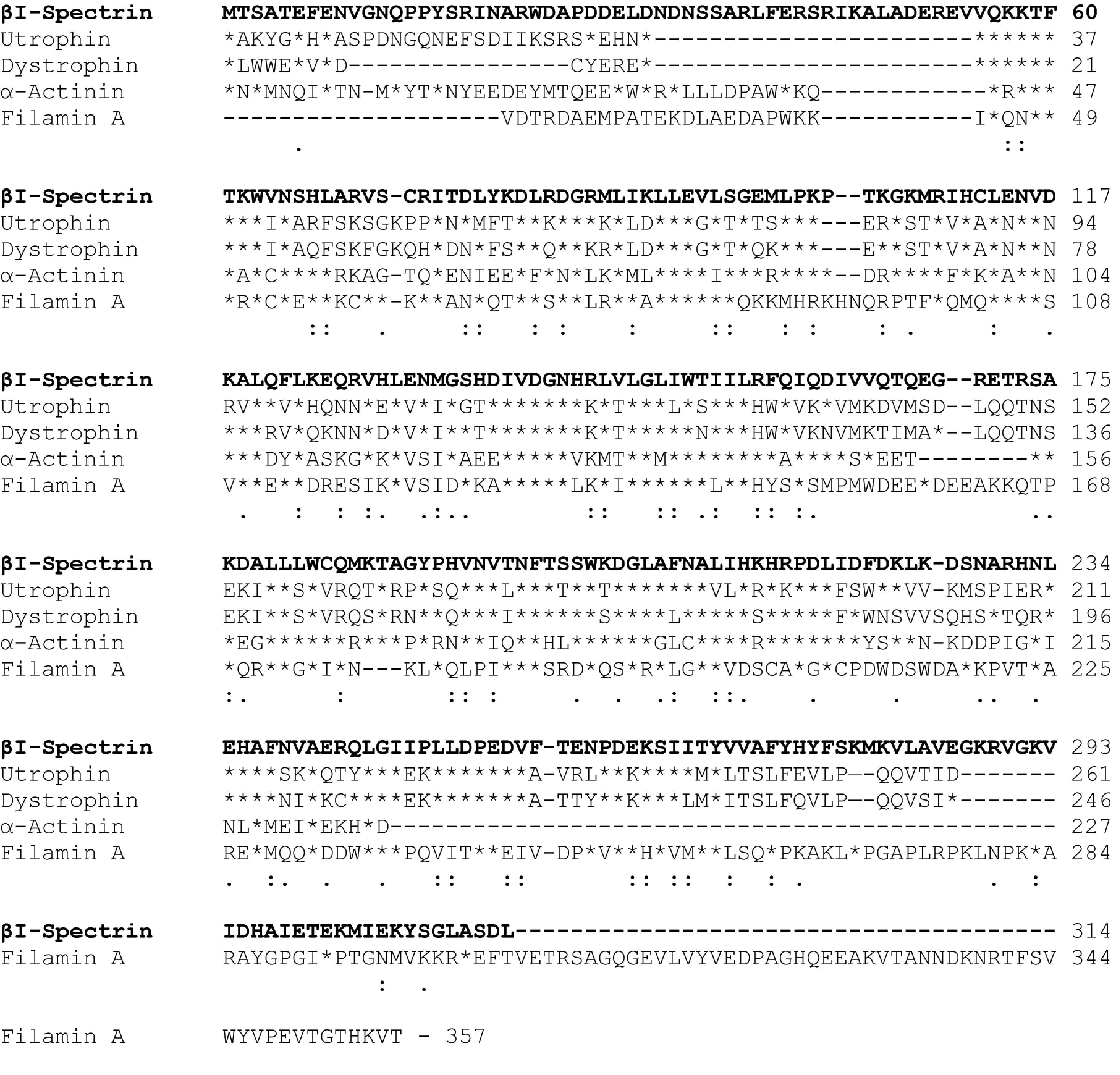

Supplement: Figure S1 — Sequence alignment of spectrin family ABDs. Although the sequences are identical to β1-spectrin at many residues (indicated by *), and very similar (indicated by :) or homologous at others (indicated by.), there are many differences. Although all share the ability to bind and bundle actin, these regions of difference are presumably responsible for the distinct activities observed in the various experiments. (TIF) [file pone.0016197.s001.tif]
